# Predictive value of cumulative SII for MACE in STEMI patients after PCI

**DOI:** 10.1097/MD.0000000000041983

**Published:** 2025-03-28

**Authors:** Weifeng Zhang, Haiyan Jia, Xingzhou Zhao, Wanqing Song, Weiwei Sun, Qianyi Wang, Yanling Li, Xiaowei Wang

**Affiliations:** a Department of Cardiology, Affiliated Hospital of Hebei University, Baoding, China; b Department of Cardiovascular Medicine, Baoding NO.1 central hospital, Baoding, China; c Yixian Hospital, Hebei Province, Baoding, China.

**Keywords:** acute ST-segment elevation myocardial infarction, inflammation, major adverse cardiovascular events, prognosis, systemic immune-inflammation index

## Abstract

The systemic immune-inflammation index (SII) has been used effectively to effectively assess the prognosis of patients with a variety of diseases. But few evidence on the relationship between SII and long-term prognosis of myocardial infarction. We thus aimed to evaluate the relationships of cumulative exposure to SII and its accumulation time course with major adverse cardiovascular events (MACE) events in patients with acute myocardial infarction after percutaneous coronary intervention. To evaluate the predictive value of SII in MACE events in patients with acute myocardial infarction. A total of 480 patients with acute ST-elevation myocardial infarction who underwent emergency coronary angiography at the Department of Cardiology, Affiliated Hospital of Hebei University from August 2022 to August 2023 were enrolled in this study. Eighteen patients were lost to follow-up, with a loss rate of 3.8%. Time-weighted cumulative SII was calculated as the weighted sum of the mean SII value for each time interval, then normalized by total exposure duration, the exposure duration was from hospitalization to 1-year follow-up. Duration of high SII exposure was defined as the duration with high SII and ranged from hospitalization to 1-year follow-up. The time course of SII accumulation was categorized by the combination of time-weighted cumulative SII < or ≥ median and SII slope. At 1-year follow-up, after adjusting for potential confounders, the time-weighted cumulative SII was divided into 2 groups. The S2 group which is above the median had a higher risk of MACE (hazard ratio, 1.090; 95% confidence interval 1.035–1.149), the high time-weighted cumulative SII group with a positive slope had a higher risk of MACE (hazard ratio, 4.096; 95% confidence interval 1.851–9.065). Long-term cumulative exposure to SII increases the risk of MACE in patients with acute ST-elevation myocardial infarction undergoing coronary angiography, and late high SII results in a higher risk of MACE events at the same time-weighted cumulative SII, underscoring the importance of late inflammation control.

## 1. Introduction

Acute myocardial infarction (AMI) is a primary cause of cardiovascular disease (CVD)-related death worldwide.^[1]^ Drug therapy and revascularization therapy have greatly improved the prognosis of patients with atherosclerotic cardiovascular disease, but the mortality rate of atherosclerotic CVD remains high,^[2]^ considering that there may still be many remaining problems unsolved. The systemic immune-inflammation index (SII) was originally applied to evaluate tumor proliferation and the pathogenesis of CVDs,^[3,4]^ and is now considered to better reflect the inflammatory state.^[5]^ SII has been used to effectively assess the prognosis of patients with a variety of diseases, including stroke^[6]^ and cancer.^[7]^ More and more recent research suggests that SII may be a potential biomarker of atherosclerosis and CVD risk. However, few studies have studied the effects of long-term exposure to SII on major adverse cardiovascular events (MACE) events of acute ST-elevation myocardial infarction. Our study stands out by using a time-weighted cumulative SII formula to dynamically track inflammation changes over 1 year. This approach differs from prior research focusing on single SII measurements, providing new insights into the temporal patterns of inflammation and their critical role in post-percutaneous coronary intervention (PCI) outcomes. This longitudinal perspective sheds light on the underexplored relationship between sustained inflammation and long-term MACE risk. In summary, it is necessary to evaluate the relationship between longitudinal SII and the prognosis of AMI. In this context, the aim of this study was to investigate the association between cumulative exposure to SII and prognosis in acute ST-elevation myocardial infarction (STEMI) patients having undergone coronary intervention.

## 2. Methods

### 2.1. Study population

The study enrolled patients with acute STEMI who were admitted to the Department of Cardiology, Affiliated Hospital of Hebei University from August 2022 to August 2023 and underwent coronary angiography.

The incidence of MACE events, including cardiac death, nonfatal myocardial infarction, ischemia-driven target revascularization, and heart failure rehospitalization, was observed at 1-year follow-up. In addition, multifactor COX analysis was performed to evaluate the risk factors of MACE events according to the occurrence or not of MACE events. Inclusion criteria: (1) patients meeting the 2019 Chinese diagnostic criteria for acute STEMI.^[8]^ (2) Patients with ≥ 1 coronary artery (left circumflex artery, left-anterior descending artery, left main artery, or right coronary artery) having a stenosis of at least 50%. (3) Patients with single-vessel disease confirmed by coronary angiography, or those with multivessel disease where non-culprit vessel stenosis is <80% and does not require further coronary intervention. Exclusion criteria: (1) patients who underwent coronary angiography or intravenous thrombolysis before being transferred from another hospital. (2) Patients with a previous coronary angiography recommendation for coronary artery bypass grafting. (3) Patients with infectious diseases within the past month, such as acute or chronic febrile respiratory infections, urinary tract infections, or gastrointestinal infections. (4) Patients with hematological diseases, autoimmune diseases, or malignancies. (5) Patients who have used biologics, glucocorticoids, immunosuppressants, or colchicine within the past 6 months. (6) Patients who have provided or received a blood transfusion. This study protocol was approved by the Ethics Committee of Hebei University Affiliated Hospital (HDFYLL-KY-2023-080), and informed consent was obtained from all patients. Chinese clinical trial registration number: ChiCTR2300072737. The registration date is June 2023. This study was supported by Medical Science Research Project of the Hebei Provincial Health Commission (20251634). Informed consent obtained during the telephone follow-up, and there is a signature during the outpatient follow-up.

### 2.2. Data collection and definitions

The demographic characteristics and lifestyle behavior factors, such as age, sex, smoking status, drinking status, as well as past medical and medication histories, were obtained through review of past medical records. Body mass index was calculated as weight (kg)/height (m)^2^. Blood was obtained from the antecubital vein early in the morning after 12 hours fast.

PCI success criteria: residual stenosis ≤ 20%, thrombolysis in myocardial infarction blood flow grade 3, and no acute re-occlusion.

MACE events included cardiogenic death, nonfatal myocardial infarction, ischemia-driven target revascularization, and heart failure rehospitalization.

### 2.3. Calculation of time-weighted cumulative SII, duration of high SII exposure and time course of SII accumulation

SII was calculated based on the patient’s complete blood count. SII = platelet count × neutrophil count/lymphocyte count was calculated based on the patient’s complete blood count. Time-weighted cumulative SII was calculated as the weighted sum of the mean SII value for each time interval, then normalized by total exposure duration, the exposure duration was from hospitalization to 1-year follow-up. The time-weighted cumulative SII formula is [(SII_in hospital_ + SII_after 1 month_)/2 × time_in hospital_ _-_ _after 1 month_ + (SII_after 1 month_ + SII_after 6 months_)/2 × time_after 1 month_ _-_ _after 6 months_ + (SII_after 6 months_ + SII_after 12 months_)/2 × time_after 6 months to after 12 months_]/time_in hospital to after 12 months_, in which SII _in hospital_, SII_after 1 month_, SII_after 6 months_ and SII_after 12 months_ indicate the results of first examination, 1 month, 6 months, and 12 months after discharge. Time _in hospital_ _-_ _after 1 month_, time_after 1 month_ _-_ _after 6 months_, time_after 6 months to after 12 months_ indicated the time intervals between 2 consecutive visits. Time_in hospital to after 12 months_ indicated the time intervals between first to fourth visits. According to time-weighted cumulative SII median, the patients were divided into S1 group (below the median group, SII < 508,226 cases) and S2 group (above the median group, SII ≥ 508,236 cases). During 1-year follow-up, SII levels changed with time, and the SII change curve had a positive slope with the increase of time, while the SII change curve had a negative slope with the decrease of time. Participants were classified using a combination of the time-weighted cumulative SII < or ≥ median and the slope of the SII curve, and were divided into 4 groups: low time-weighted SII with negative slope (reference group), high time-weighted SII with negative slope, low time-weighted SII with positive slope, and high time-weighted SII with positive slope.

### 2.4. Primary endpoint

The incidence of cardiac MACE events was observed during 1-year follow-up.

### 2.5. Statistical analysis

SPSS 26.0 was used for statistics, and measurement data conforming to normal distribution were presented as x ®±s. Comparison between groups was performed by *T* test or Mann–Whitney rank sum test. The statistical data were expressed as a percentage and were tested by χ^2^. Receiver operating characteristic (ROC) analysis determines the best prediction threshold for MACE events. The Kaplan–Meier curve was constructed to compare the incidence of MACE events between different groups, and was tested with log rank. The influencing factors of MACE events were analyzed by COX proportional risk regression model. Test level α = 0.05.

## 3. Results

### 3.1. Baseline characteristics

Among the 480 patients included, 377 males and 103 females, aged 36 to 79 (58.8 ± 10.0) years, were followed up for 1 year, and 18 cases were lost to follow-up, with a loss rate of 3.8%. 462 patients were returned by telephone. There were significant differences in history of ischemic stroke, fasting blood glucose, triglyceride, homocysteine, SII and left ventricular ejection fraction between S1 and S2 groups (*P* < .05). Compared with S1 group, S2 group had a higher proportion of ischemic stroke history (χ^2^ = 10.656, *P* < .001), higher fasting blood glucose (t = -4.431, *P* < .001), higher SII (Z = -0.042, *P* < .001), and lower left ventricular ejection fraction (t = 12.428, *P* < .001) (Table 1).

**Table 1:** Baseline characteristics of participants according to median of time-weighted cumulative SII.

Item	Group S1 (226)	Group S2 (236)	t/*χ*^2^	*P*-value
Age (year)	58.9 ± 9.9	58.7 ± 10.1	0.641	.424
Sex [n (%)]	185 (81.9)	183 (77.5)	1.327	.249
BMI	25.1 ± 2.9	25.7 ± 3.2	0.174	.677
Smoking history [n (%)]	155 (68.6)	171 (72.5)	0.834	.361
Drinking history [n (%)])]	74 (32.7)	44 (18.6)	12.067	.001
Family history of CHD [n (%)]	26 (11.5)	37 (15.7)	1.707	.191
Hypertension [n (%)]	144 (63.7)	134 (56.8)	2.318	.128
Diabetes [n (%)]	58 (25.7)	78 (33.1)	3.033	.822
History of ischemic stroke [n (%)]	12 (5.3)	34 (14.4)	10.656	**.001**
Door to ballon time (h)	10.6 ± 8.6	10.0 ± 7.1	2.881	.090
WBC (*10^9^/L)	6.4 ± 1.8	7.6 ± 1.9	2.728	.099
Serum creatinine (µmol/L)	71.7 ± 14.9	71.4 ± 15.9	0.703	.074
Uric acid (µmol/L)	337.8 ± 93.2	321.1 ± 68.8	0.487	.485
FGP (mmol/L)	5.9 (5.2, 8.2)	7.0 (5.0, 12.1)	-4.431	**<.001**
Peak troponin-I (ng/mL)	11.1 (5.5, 33.5)	12.3 (6.1, 40.1)	-0.875	.382
Total cholesterol (mmol/L)	4.5 ± 1.1	4.3 ± 1.1	2.173	.141
LDL-C (mmol/L)	2.9 ± 0.9	2.6 ± 0.8	0.000	.999
Triglyceride (mmol/L)	2.0 ± 0.7	1.9 ± 0.9	6.755	**.010**
BNP (pg/mL)	48.1 (22.5, 116.0)	52.9 (27.1, 111.1)	0.000	.842
HCY (µmol/L)	12.3 (10.3, 15.1)	11.7 (9.2, 14.1)	-3.543	**.000**
CRP (µmol/L)	5.1 ± 2.0	5.6 ± 2.1	3.605	.058
SAA (µmol/L)	9.1 (6.3, 11.8)	9.5 (7.2, 11.4)	-0.199	.842
SII	384.6 (282.6, 453.2)	674.8 (584.2, 898.8)	-0.042	**<.001**
LVEF (%)	61.8 ± 5.3	60.8 ± 8.7	12.428	**<.001**
Aspirin [n (%)]	226 (100)	236 (100)	1.000	NS
ADP receptor antagonists [n (%)]	226 (100)	236 (100)	1.000	NS
Statin [n (%)]	220 (97.3)	226 (95.8)	0.865	.352
Beta-bloker [n (%)]	199 (88.1)	206 (87.3)	0.062	.803
ACEI/ARB/ARNI [n (%)]	197 (87.2)	204 (86.4)	0.054	.817

Bold values indicate statistically significant difference.

ACEI = angiotensin-converting enzyme inhibitor, ARB = angiotensin II receptor blocker, ARNI = angiotensin receptor-neprilysin inhibitor, BMI = body mass index, BNP = B-type natriuretic peptide, CHD = coronary heart disease, CRP = C-reactive protein, FGP = fasting glucose plasma, HCY = homocysteine, LDL-C = low-density lipoprotein cholesterol, LVEF = left ventricular ejection fraction, SAA = serum amyloid A, SII = the systemic immune-inflammation index, WBC = white blood cell.

### 3.2. Comparison of coronary angiography and interventional therapy between S1 group and S2 group

There was no significant difference between S1 group and S2 group in the location of diseased vessels, number of diseased vessels, postoperative thrombolysis in myocardial infarction 3 grade and number of stents (*P* > .05) (Table 2).

**Table 2:** Comparison of coronary angiography and interventional therapy between S1 group and S2 group [n (%)].

Item	Group S1 (226)	Group S2 (236)	*χ* ^2^	*P*-value
Infarct-related coronary artery			0.062	.109
Left main	7 (3.1)	10 (4.2)		
Left-anterior descending	90 (39.8)	86 (36.4)		
Left circumflex	56 (24.8)	42 (17.8)		
Right	73 (32.3)	98 (41.5)		
Multivessel coronary disease			0.952	.621
1	93 (41.2)	99 (41.9)		
2	66 (29.2)	60 (25.5)		
3	67 (29.6)	77 (32.6)		
Pre-PCI TIMI flow grade 0	66 (29.2)	71 (30.1)	0.043	.836
Thrombus aspiration	25 (11.1)	24 (10.2)	0.097	.755
Pro-PCI TIMI flow grade 3	216 (95.6)	223 (94.5)	0.287	.592
Number of stents			0.584	.747
0	23 (10.2)	29 (12.3)		
1–2	191 (84.5)	196 (83.1)		
≥3	12 (5.3)	11 (4.7)		

TIMI = thrombolysis in myocardial infarction.

### 3.3. Secondary prevention of risk factors after discharge in groups S1 and S2

There was no significant difference between S1 group and S2 group in fasting blood glucose, low-density lipoprotein cholesterol, blood pressure control, and medication compliance at 6 months after discharge and 12 months after discharge (*P* > .05) (Tables 3 and 4).

**Table 3:** Fasting blood glucose, low-density lipoprotein cholesterol, and blood pressure meeting rates in groups S1 and S2 [n (%)].

	FPG (mmol/L)		LDL-C (mmol/L)		BP	12-month
	6-month	12-month	6-month	12-month	6-month	
Group S1 (226)	203 (89.8)	194 (85.8)	162 (71.7)	170 (75.2)	182 (80.5)	169 (74.8)
Group S2 (236)	207 (87.7)	196 (83.1)	176 (74.6)	184 (78.0)	189 (80.1)	176 (74.6)
χ^2^	0.515	0.683	0.493	0.486	0.015	0.003
*P*	.473	.409	.483	.486	.904	.960

BP = blood pressure, FGP = fasting glucose plasma, LDL-C = low-density lipoprotein cholesterol.

**Table 4:** Compliance medicine comparison [n (%)].

	Aspirin		ADP receptor antagonists		Statin		Beta-bloker		ACEI/ARB/ARNI	
	6-month	12-month	6-month	12-month	6-month	12-month	6-month	12-month	6-month	12-month
Group S1 (226)	225 (99.6)	224 (99.1)	220 (97.3)	218 (96.5)	217 (96.0)	201 (93.1)	217 (96.0)	199 (88.1)	201 (88.9)	198 (87.2)
Group S2 (236)	234 (99.2)	233 (98.7)	232 (98.3)	231 (97.9)	224 (94.9)	213 (90.3)	229 (97.0)	202 (85.6)	203 (86.0)	201 (85.2)
χ^2^	0.293	0.161	0.502	0.853	0.323	1.149	0.357	0.610	0.897	0.410
*P*	.588	.688	.478	.356	.570	.284	.550	.435	.344	.522

ACEI = angiotensin-converting enzyme inhibitor, ARB = angiotensin II receptor blocker, ARNI = angiotensin receptor-neprilysin inhibitor.

### 3.4. Comparison of primary endpoint events between the 2 groups

During 1-year follow-up, a total of 52 patients had MACE events, and the incidence of MACE events in S2 group was higher than that in S1 group (*χ*^2^ = 4.797, *P* = .029) (Table 5).

**Table 5:** primary endpoint events [n (%)].

	Group S1 (226)	Group S2 (236)	*χ^2^*	*P*-value
MACE	18 (8.0)	34 (14.4)	4.797	**.029**

Bold value indicates statistically significant difference.

### 3.5. Kaplan–Meier curve of MACE events after combining time-weighted cumulative SII < or ≥ median array and with SII slope

**Figure 1.**
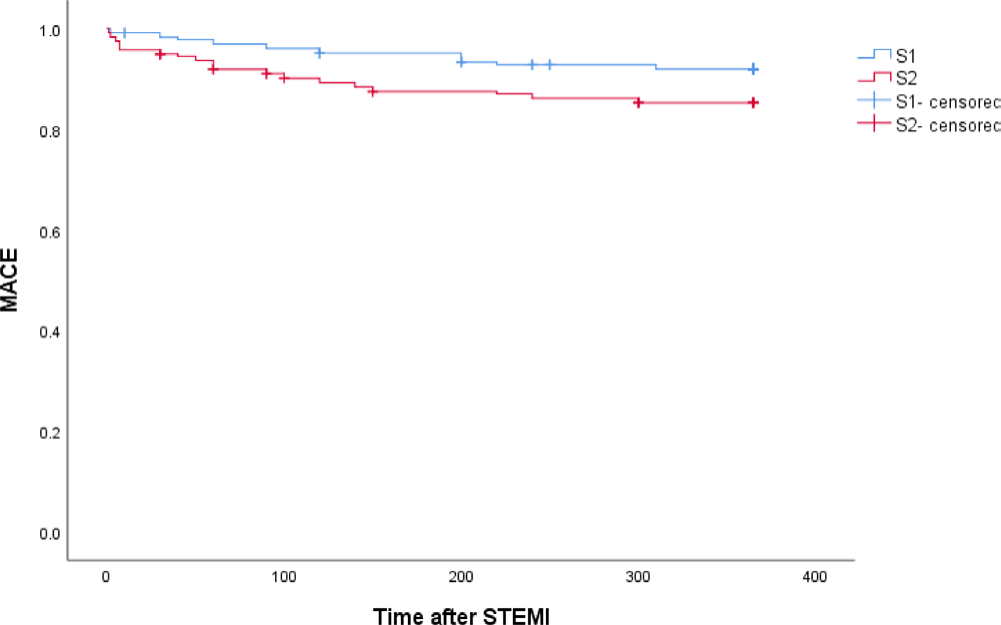
The survival curves of MACE events between S1 and S2 groups. MACE = major adverse cardiovascular events.

**Figure 2.**
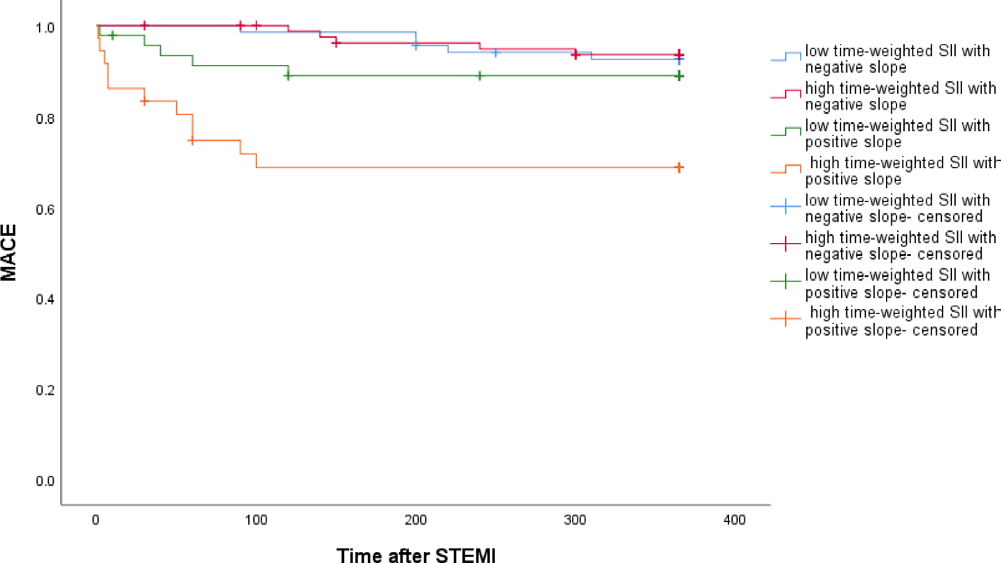
Survival curve analysis of MACE events after the combination of time-weighted cumulative SII < or ≥ median array and SII slope. MACE = major adverse cardiovascular events, SII = systemic immune-inflammation index.

The survival curves of MACE events between S1 and S2 groups were statistically significant (Log Rank = 5.253, *P* = .022). Higher time-weighted SII survival with positive slope was lower than that of other groups, and the difference was statistically significant (Log Rank = 42.695, *P* < .001) (Figs. 1 and 2).

### 3.6. We selected confounding factors based on clinical relevance and a univariate analysis criterion of *P *< .1

After multifactor correction, there was a statistically significant difference between time-weighted cumulative SII < and ≥ median. A statistically significant difference was observed between high time-weighted cumulative SII with positive slope and low time-weighted cumulative SII with negative slope (Tables 6 and 7).

**Table 6:** Univariate analysis for MACE events.

Item	MACE	
	HR (95% CI)	*P*
Age	0.980 (0.944, 1.018)	.297
Sex	4.482 (2.077, 9.670)	<.001
Smoking history	0.366 (0.110, 1.221)	.102
History of stroke	1.653 (0.570, 4.797)	.355
FGP	1.052 (0.986, 1.121)	.123
Triglyceride	1.094 (0.843, 1.420)	.500
HCY	0.991 (0.949, 1.034)	.664
SII	1.924 (1.807, 3.408)	.025
EF	1.096 (1.019, 1.179)	.014
CRP	1.076 (1.053, 1.099)	<.001

CRP = C-reactive protein, FGP = fasting glucose plasma, HCY = homocysteine, LVEF = left ventricular ejection fraction, SII = the systemic immune-inflammation index.

**Table 7:** Correlation analysis between S1 and S2 groups, combined with the slope of SII curve and MACE events.

	MACE					
	HR (95% CI)^a^	*P*	HR (95% CI)^b^	*P*	HR (95% CI)^c^	*P*
Time-weighted cumulative SII						
S1	Reference					
S2	1.924 (1.807, 3.408)	.025	1.116 (1.052, 1.183)	<.001	1.100 (1.044, 1.158)	**.046**
Time-weighted cumulative SII combined slope						
Low time-weighted cumulative SII with negative slope	Reference					
High time-weighted cumulative SII with negative slope	0.856 (0.356, 2.057)	.729	0.890 (0.370, 2.138)	.794	0.778 (0.320, 1.894)	.580
Low time-weighted cumulative SII with positive slope	1.607 (0.669, 3.861)	.289	1.926 (0.796, 4.656)	.146	1.351 (0.554, 3.290)	.508
High time-weighted cumulative SII with positive slope	5.432 (2.570, 11.483)	<.001	4.527 (2.132, 9.612)	<.001	4.601 (2.112, 8.022)	**<.001**

Model a: uncorrected; Model b: adjust for age and sex; Model c: adjusted for gender, age, personal history of diabetes mellitus, hypertension or ischemic stroke, fasting blood glucose, triglyceride, HCY, CRP, left ventricular ejection fraction, 6-month fasting blood glucose compliance rate, 6-month low-density lipoprotein cholesterol compliance rate, 6-month blood pressure compliance rate, 6-month medication compliance (including aspirin, ADP receptor antagonists, statins, beta blockers, ACEI/ARB/ARNI), 12-month fasting blood glucose compliance rate, 12-month low-density lipoprotein cholesterol compliance rate, 12-month blood pressure compliance rate, 12-month compliance status (including aspirin, ADP receptor antagonists, statins, beta blockers, ACEI/ARB/ARNI). Bold values indicate statistically significant difference.

HR = hazard ratio, SII = the systemic immune-inflammation index.

### 3.7. ROC curve

**Figure 3.**
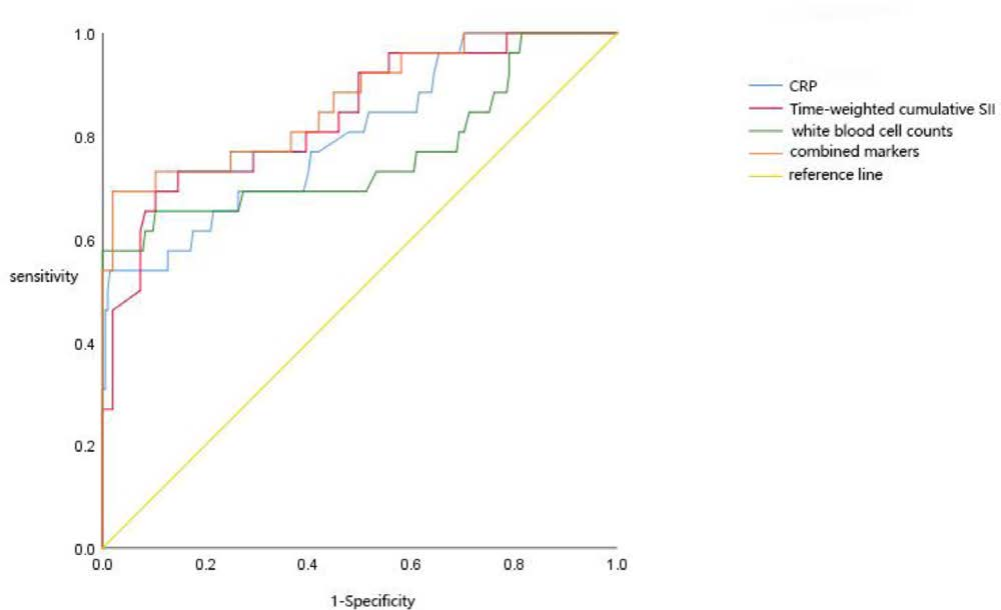
ROC curve of MACE events predicted by single inflammatory markers and combined markers. MACE = major adverse cardiovascular events, ROC = receiver operating characteristic.

Time-weighted cumulative SII, C-reactive protein, and white blood cell counts independently and jointly predicted the ROC curve of MACE events (Table 8, Fig. 3). ROC curve results of time-weighted cumulative SII for MACE events showed that the area under curve of time-weighted cumulative SII was 0.839 (95% confidence interval [CI] 0.752 to 0.927; *P* < .001), the best cutoff value was 941, the sensitivity was 63.8%, and the specificity was 88%. The combined prediction results were better, with the specificity 0.692, sensitivity 0.08, and area under the curve 0.867.

**Table 8:** ROC analysis of time-weighted cumulative SII, CRP, and white blood cell counts independently and jointly predicted MACE events.

Test variable	AUC	Standard error	*P*-value	95% CI
C-reactive protein	0.801	0.050	**<.001**	0.702–0.900
Time-weighted cumulative SII	0.839	0.045	**<.001**	0.752–0.927
White blood cell counts	0.765	0.064	**<.001**	0.638–0.891
Combined indicator	0.867	0.043	**<.001**	0.782–0.952

Bold values indicate statistically significant difference.

AUC = area under curve.

### 3.8. Subgroup analysis

**Figure 4.**
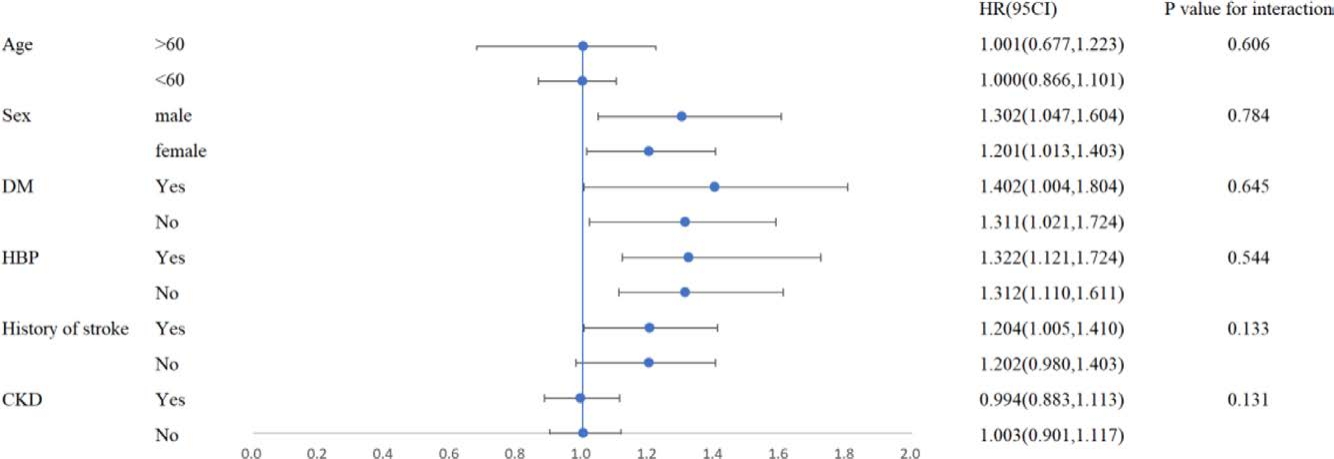
Subgroup analysis of MACE events. MACE = major adverse cardiovascular events.

To assess the potential heterogeneity of SII on MACE, we performed subgroup analysis stratified by the following factors: age (≥60 years vs <60 years), sex (male vs female), presence of hypertension, diabetes, history of ischemic stroke, and chronic kidney disease. On the subgroup analysis, a significant association was found between SII and the occurrence of MACE in patients with a history of ischemic stroke. However, no significant relationship was observed between SII and MACE in patients without a history of stroke (Fig. 4).

## 4. Discussion

In this study, a large proportion of the enrolled patients did not reach the standard of lower density lipoprotein at admission, and the compliance with statin application was still good after discharge. However, after 6 months of follow-up, about 1/4 of the patients still failed to reach the standard of lower density lipoprotein, which was firstly considered to be related to the risk of remaining lipids after medium-dose statin treatment used in China. Statins combined with non-statins have become the main means of lipid-lowering treatment, but the acceptance and utilization rate of combined drug use by outpatient doctors and patients need to be improved, and the substandard low-density lipoprotein may be related to the unreasonable diet and bad lifestyle of some patients, so we should strengthen the strict control of blood lipids and strengthen the popularization of knowledge of combined drug use in the future. After 6 months, there was no statistically significant difference between the fasting blood glucose groups, but 14% to 17% of the patients did not meet the blood glucose standard, so it is necessary to strengthen the attention of outpatient doctors and patients to meet the blood glucose standard. After 6 months of follow-up, the poor rate of patients’ blood pressure reaching the standard may be related to medication compliance, and outpatient doctors may not be effective in adjusting drugs only by measuring blood pressure in the outpatient department. Therefore, the implementation of ambulatory blood pressure monitoring in the outpatient department or self-measuring blood pressure at home should be strengthened to improve the blood pressure reaching the standard rate.

In this study, the incidence of MACE was 11.3% in 462 STEMI patients 1 year after PCI, so it is of great significance to predict the risk of MACE early. The CANTOS trial^[9–12]^ revealed the inflammation-based pathogenesis of coronary atherosclerosis. Canazumab (a targeted inhibitor of inflammatory cytokine interleukin-1β) reduced hypersensitive C-reactive protein in patients with AMI without affecting lipid levels, reducing major MACE events by 15% in patients receiving standard secondary prevention. Thus, it opens the era of targeted anti-inflammatory therapy for coronary atherosclerotic diseases. Therefore, prognostic markers of patients with AMI should fully reflect the lipid metabolism and inflammatory state of the body. At the same time, a large number of studies have shown that systemic inflammation plays a central role in CVD. Neutrophils and platelets, which play a role in the process of chronic inflammation, and lymphocytes, which play a role in the subsequent immune response, are important factors in the occurrence and development of atherosclerosis.^[13–15]^ Inflammation and immunity-based prognostic scores such as neutrophil/lymphocyte ratio, platelet lymphocyte ratio, and established pro-inflammatory biomarkers (white blood cell and C-reactive protein levels) have been shown to predict mortality after cardiac surgery.^[16,17]^ Recently, as a comprehensive indicator of neutrophil, platelet, and lymphocyte levels, SII has become an important prognostic factor for CVDs.^[18–21]^ High SII scores have previously been shown to be associated with poor prognosis in patients with coronary atherosclerosis.^[22,23]^ This study analyzed the relationship between the dynamic course of SII and the prognosis of STEMI patients, and the results suggested that high levels of SII were independently associated with long-term poor prognosis in acute SETMI patients undergoing PCI, and that higher levels of SII in the late stage may lead to poorer prognosis compared with higher levels of SII in the early stage, suggesting the importance of controlling inflammatory indicators in the later stage.

SII is a novel biomarker that, in essence, is calculated from the count of 3 key immune cells (lymphocytes, neutrophils, and platelets) and therefore reflects the systemic inflammatory state and acts as a routine measure of systemic inflammation. It is well known that immune cells are involved in cardiac damage and repair,^[24–26]^ and many studies have shown that SII is an independent predictor of poor outcomes in STEMI outcomes, such as death, arrhythmia, and stent thrombosis. From a mechanistic perspective, activated neutrophils release a variety of proteolytic enzymes, such as myeloperoxidase and elastase, resulting in myocardial damage.^[27,28]^ In contrast, lymphocytes are an early marker of physiological stress,^[29,30]^ representing a regulated inflammatory process that suppresses excessive immune responses and limits myocardial damage. Regarding platelets, in patients with systemic inflammation, the disease can be aggravated by platelet aggregation, adhesion, recruitment, and release.^[31]^ Activated platelets release a large number of pro-inflammatory cytokines and cytokines that cause thrombosis or interact with other white blood cells to aggravate atherosclerosis and plaque instability. This is often associated with harmful cardiovascular outcomes.^[32]^ In a retrospective cohort study of 314 elderly non-STEMI patients, Orhan et al proved that the higher the level of SII, the higher the in-hospital mortality and long-term mortality, and the above results corrected for age, diabetes, hypertension, heart failure, and other indicators.^[33]^ In addition, in a study by Li et al, in 1701 patients with acute coronary syndrome who underwent PCI, SII was shown to be an independent predictor of all-cause death, nonfatal ischemic stroke, and nonfatal myocardial infarction. In addition, adding SII to GRACE risk score can significantly improve the predictive value of the latter.^[34]^ Consistent with previous studies, we found that higher SII levels remain an independent risk factor for long-term outcomes in acute SETMI patients undergoing PCI. The association of SII with LVEF impairment and extensive myocardial necrosis has been revealed, which may partially explain the adverse prognostic effects of high SII, as decreased LVEF and elevated cTnI levels are known to be harmful. On the other hand, as an emerging indicator of pre-thrombotic activity, high SII levels may indicate excessive thrombotic burden, which is also considered to be an important risk factor for non-reflow and poor survival after STEMI.^[34]^

Emerging studies have analyzed that certain hypoglycemic agents, such as sodium-glucose co-transporter 2 inhibitor (SGLT2-I), improve survival in STEMI patients, in part due to their anti-inflammatory properties.^[35,36]^ A multicenter international observational study continuously included 583 patients with AMI complicated with diabetes. Compared with patients receiving other oral hypoglycemic drugs, patients with AMI complicated with type 2 diabetes receiving SGLT2-I treatment had significantly reduced inflammatory response and smaller infarct size, which had nothing to do with blood glucose metabolism control.^[35]^ The EMPA-REG OUTCOME trial randomized to englaglizin in patients with confirmed CVD compared with placebo reported a 14% reduction in major composite outcomes of cardiovascular death, nonfatal myocardial infarction, and nonfatal stroke, and a > 30% reduction in cardiovascular death. However, the cardioprotective mechanism of SGLT2-I in coronary artery disease remains unclear. Studies have confirmed that it can play a role by increasing osmotic diuresis, sodium excretion, reducing the front and back load of the heart, and inhibiting the sodium–hydrogen exchanger of the heart, which may involve multiple processes that directly or indirectly improve myocardial performance, including improving proximal renal tubular dysfunction.^[37–39]^ It also has an impact on inflammatory processes, extracellular mechanisms and fibrosis-related molecular processes.^[40]^

SII integrates the count of neutrophils, lymphocytes, and platelets to comprehensively reflect the systemic immune-inflammatory status. Compared to single inflammatory markers, SII offers a more dynamic and accurate assessment of inflammation, with higher sensitivity and predictive ability. It is closely associated with the long-term prognosis of STEMI patients, and its predictive accuracy is further improved when combined with other clinical factors. Additionally, SII is simple to calculate and cost-effective, making it suitable for routine clinical practice, especially in resource-limited settings. By dynamically tracking inflammation levels using the time-weighted cumulative SII formula, particularly during the recovery phase after PCI, it provides valuable insights into long-term prognosis. Persistent high SII or an increasing trend may indicate a higher risk of complications, helping physicians adjust treatment plans. Integrating SII with AI and big data analysis can further optimize clinical decision-making, support personalized treatment, reduce the risk of MACE, and improve overall patient outcomes.

## 5. Conclusion

We demonstrate that high SII is an independent risk factor for poor long-term outcomes in acute STEMI patients undergoing PCI. Despite the study’s limitations, our results hold significant clinical value. Future research should focus on validating SII’s role in risk stratification and its ability to predict long-term complications through large-scale, multicenter studies. Combining SII with other biomarkers can enhance clinical risk assessment accuracy. Moreover, evaluating the impact of anti-inflammatory therapy on SII may shed light on its potential for improving long-term prognosis. Investigating the dynamic changes in SII and their relation to prognosis will further highlight its predictive value in long-term follow-up. Lastly, developing a clinical decision support system that integrates AI and big data can help optimize treatment plans and improve patient outcomes.

## 6. Limitations and prospects

The study also had some limitations. First, this study is a single-center retrospective design with a small sample size and a short follow-up period, which limits the generalizability of the findings. Future multicenter studies with larger sample sizes are needed to confirm and expand the applicability of these results. Second, while our study excluded infectious diseases, hematological disorders, and autoimmune diseases, excluded patients using drugs that affect blood counts, and corrected for the effects of C-reactive protein, we still could not eliminate the effects of some unmeasured confounders. At the same time, we hope to design prospective study and expanding the sample size to reduce the impact of unmeasured confounders.

## Author contributions

**Conceptualization:** Weifeng Zhang.

**Data curation:** Weifeng Zhang, Haiyan Jia, Xingzhou Zhao.

**Formal analysis:** Weifeng Zhang, Haiyan Jia, Wanqing Song.

**Funding acquisition:** Haiyan Jia.

**Investigation:** Xiaowei Wang, Weiwei Sun.

**Methodology:** Weifeng Zhang, Yanling Li, Xiaowei Wang, Qianyi Wang.

**Project administration:** Haiyan Jia.

**Resources:** Yanling Li, Haiyan Jia.

**Software:** Haiyan Jia.

**Supervision:** Yanling Li, Haiyan Jia.

**Validation:** Xiaowei Wang.

**Visualization:** Yanling Li.

**Writing – original draft:** Weifeng Zhang, Haiyan Jia, Xiaowei Wang.

**Writing – review & editing:** Weifeng Zhang, Haiyan Jia, Xiaowei Wang.
